# Prevalence and associated factors for rural households food insecurity in selected districts of east Gojjam zone, northern Ethiopia: cross-sectional study

**DOI:** 10.1186/s12889-020-8220-0

**Published:** 2020-02-07

**Authors:** Amare Wubishet Ayele, Mulusew Kassa, Yenesew Fentahun, Hayimro Edmealem

**Affiliations:** 1 Department of Statistics, College of Natural and Computational Science, Debre Markos University, P.O. Box 269, Debre Markos, Ethiopia; 2 Department of Plant Science, College of Agriculture and Natural Resources, Debre Markos University, P.O. Box 269, Debre Markos, Ethiopia

**Keywords:** East Gojjam zone, Ethiopia, Food insecurity, Partial proportional odds model, Rural household

## Abstract

**Background:**

Food insecurity is a pressing social and public health issue that varies in degree and impact on individuals and social groups, requiring immediate attention for policymakers and decision-makers. This study was conducted to identify the prevalence and associated factors of food insecurity of rural households particularly in the Shebel Berenta and Machakel districts of East Gojjam zone.

**Methods:**

A cross-sectional study design was conducted, in the fall of March 2017 among 504 households. Households are selected using a systematic sampling technique through multistage cluster sampling technique (two stage cluster sampling). The data were collected using a structured interviewer-administered questionnaire covering a range of topics including 18 core food security modules (CFSM) question series, socioeconomic, demographic and related variables. Multivariable Partial proportional odds model (PPOM) was employed to identify the factors associated with food insecurity in rural households.

**Result:**

Of a total of 504 households considered in the study, 54 (10.71%) were highly food secure, 75 (14.88%) were marginally food secure, 157 (31.15%) were low food secure, and 218 (43.25%) were severely food insecure. District (Machakel) (AOR = 3.28 95% CI: 1.73, 6.24), household head education status, illiterate (AOR = 113.4, 95% CI:7.02,1832.02), read and write (AOR = 169.29, 95%CI:11.64, 2461.39), and elementary completed (AOR = 119.75,95%CI:8.43,1700.74), agro-ecological zone, Woina Dega (AOR = 0.0021,95% CI: 0.00009,0.0514), Dega (AOR = 0.0323, 95%CI: 0.002, 0.5209), family size (AOR = 1.18, 95%CI: 1.01, 1.36), landholding (AOR = 0.767, 95% CI: 0.605, 0.972), TLU (AOR = 0.151, 95% CI: 0.0716, 0.3189), access to toilet (no) (AOR = 7.63, 95% CI: 1.459, 39.78), practicing irrigation (yes) (AOR = 0.121, 95% CI: 0.037, 0.38), loan (no) (AOR = 2.83, 95% CI:1.36, 5.89), access to energy, government electric (AOR = 0.468, 95% CI: 0.23, 0.94), solar panels (AOR = 0.45, 95% CI: 0.25, 0.79), soil fertility, moderate (AOR = 0.28, 95% CI: 0.12, 0.87), fertile (AOR = 0.15, 95% CI: 0.032, 0.72) were significant associated food insecurity factors in the study area.

**Conclusion:**

In this study, a high prevalence of food insecurity and various associated food insecurity factors have been identified in the study area. Thus, the concerned stockholders should intervene in food insecure households via different irrigation practices and by considering household size, community-based household head education, and landholding in hectare.

## Background

Food insecurity is a situation where the availability of sufficient quantity of affordable, nutritionally appropriate and secure food is restricted or uncertain, or the socially acceptable capacity to obtain appropriate food is limited [1, 2]. Food insecurity is characterized by low food intake, intermittent access to food, and vulnerability to a livelihood strategy that generates adequate food in good times but is not resilient to shocks [3].

Globally, the number of people facing chronic food deprivation has risen from around 804 million in 2016 to nearly 821 million in 2017. Most of the world’s hungry people live in developing countries, where 12.9% of the population is undernourished. Asia is the continent with two-third of the total hungry people. But currently, this percentage has- fallen in South Asia, and slightly increased in West Asia. Sub - Saharan Africa is the region with the highest prevalence of hunger, with one in four people undernourished [4, 5].

Household food insecurity affects food intake and may prevent an individual from consuming enough of the right kinds of nutrients to support and maintain health. Adults with food insecurity report bad health, including mental, physical and oral health, and chronic conditions such as diabetes, heart disease, hypertension, anxiety, and fibromyalgia [6, 7]. Food insecurity adds a significant premium to the health care system by impacting people’s ability to handle chronic health problems [8]. Besides, children suffering from hunger are more likely to have worse health, suggesting that repeated episodes of hunger in children over time lead to higher risks of chronic diseases and asthma [9].

Sub-Saharan Africa has hardly been confronted with the food crisis [10], and food insecurity is still a major concern. The underlying causes of food crisis and insecurity include both short-term and long- term factors such as rising food prices and food riots, climate change and weather vagaries, political upheavals and conflicts, extreme poverty and lack of agricultural productivity [11]. Food supply is obviously insufficient in sub-Saharan Africa, as production in agriculture is low-yield and has been declining [12]. Due to low agricultural production, subsidies and trade protection for their farmers are still provided from developed countries [13].

Over the last two decades, Ethiopia has made significant gains in development by reducing poverty and increasing investment in basic social services. In the face of a number of attempts so far made in Ethiopia to improve overall food insecurity, this is still a major problem [14]. In 2017, the food security scenario in Ethiopia deteriorated dramatically, with the estimated food insecurity, population rising from 5.6 million in December 2016 to 8.5 million in August 2017, due to severe drought, conflict instability and crop disease among the primary drivers [15]. The livelihood of most Ethiopians depends on agriculture, especially among the poor rural population. Approximately 84% of the country’s labor force takes agriculture in Ethiopia, which is the largest contributor to economic growth and the most important sector of the economy. Although it is a country with significant agricultural potential due to its water resources, fertile land areas and a large labor pool, this potential is largely unutilized [3].

Food insecurity is currently a critical social issue that requires immediate attention from policymakers and other decision-makers [16–18]. Food insecurity is a pressing social and public health issue that varies in degree and impact on individuals and social groups. For this reason, it is critical to understand how food insecurity patterns appear across different demographic and socio-economic issues to meet specific needs by implementing appropriate policy programs and other initiatives [19, 20].

Reducing food insecurity in Ethiopia as a result of events such as population growth, food prices, and recurrent drought continues to be a major public policy challenge, and one that is complicated by lack of information on the location, severity, and causes of food insecurity. Such information is needed for properly targeted assistance, assess progress, and develop appropriate interventions to help those in need [21]. In addition, food insecurity is a multidimensional concept, experienced differently by different types of households and population groups, and it is a complex issue that may not be fully captured by a one dimensional item response model, especially as it will be used to track food insecurity over time, across different surveys, and for different subpopulations [22, 23].

So far, in different areas of the globe [24–27], and also in Ethiopia [28–30], various study works have been carried out on food insecurity. Most of the previous food insecurity studies focused primarily on children and single-parent households and also used a single indicator to measure the food insecurity situation. Moreover, different approaches are commonly used in the national survey to assess food insecurity, namely the FAO method, household expenditure survey method, dietary intake assessment, and anthropometry method [31, 32].

The dietary intake assessment approach is criticized for missing nutrient adequacy [33] and lack of identified calorie intake and replacement impact due to increased income [34]. The FAO method is disapproved for not being specific, it focuses on food insecurity risk at the national level [31]. Likewise, the anthropometry approach is criticized because it only measures the effects of food insecurity; and the household expenditure survey method is slated because it only measures the amount of food available but not necessarily the amount of food consumed and makes it difficult to quantify the amount of food consumed outside the home. This article deviates from the previous approach of studying food insecurity by using continuous food security measure that incorporates questions on the core food security module and multiple indicators. This approach directly measures the phenomenon of interest based on the experience of food insecurity as perceived by the vulnerable group, and it captures not only the physical but also the psychosocial dimensions of food insecurity [31].

Furthermore, to the best of our knowledge, food insecurity is less clearly documented in the study area. Moreover, the statistical methodology used in the related food insecurity literature was more qualitative and could not demonstrate the magnitude of the severity of food insecurity and could not explicitly specify the associated factors. Therefore, this study aims to evaluate the prevalence and associated food insecurity factors of households in selected districts of East Gojjam Zone, Northern Ethiopia via Partial proportional odds model.

## Methods

### Study area and design

This study was conducted on two randomly selected districts, particularly Machakel and Shebel Berenta, in the East Gojjam zone. In this study, the household-based cross-sectional survey was conducted to investigate the prevalence and associated food insecurity factors, and the study population considers all households in the study area at the survey time.

### Data collection and measurement

For this study, primary data was used. Data were collected using a structured interviewer-administered questionnaire that was adopted from different standard questionnaires in the fall of March 2017. The questionnaire covered a range of topics including 18 items core food security module (CFSM) question series, socioeconomic, demographic and related variables. For the most part, a pretest or pilot study was conducted to evaluate the questionnaire’s clarity and suitability, and to obtain different estimates for sample size determination. After providing the necessary training for data collectors and supervisors on confidentiality, data collection process, study objectives, and their importance, the actual data collection process was assisted by eight data collectors and two supervisors. Before data collection, permission to conduct the study was obtained from Debre Markos University’s Directorate of Research and Technology Transfer.

The response variable for this study was food insecurity status of households with status of highly food secure, marginally food secure (mildly food insecure), low food secure (moderately food insecure), and severely food insecure, which has natural ordering pattern with the degree of severity increases [35, 36]. A household is classified into one of the food security status-level categories based on household’s scale score on the food security scale using the set of CFSM indicator questions (Additional file 1). Socio-economic, demographic, environmental and institutional predictors related to the characteristics of the household were adopted from different pieces of literature and based on the economic theory of food insecurity [37–42].

### Operational definition

**Food insecurity** -is a state in which consistent access to adequate food is limited at times throughout the year due to a lack of money and other resources. It refers to the social and economic problem, lack of food due to a resource or other constraints, not voluntary fasting or dieting, or because of illness, or for other reasons [43, 44].

**Food security-** is a state in which all people, at all times, have physical and economic access to sufficient, safe and nutritious food to meet their dietary needs and food preferences for an active and healthy life [45].

**Highly Food secure (HFS)** – households that show no or minimal evidence of food insecurity.

**Marginally food secure (MFS)** – households that have an evident in household members’ concerns about the adequacy of the household food supply and adjustments to household food management, including reduced quality of food and increased unusual coping patterns (Food insecure without hunger).

**Low food secure (LFS)** - households who show food intake for adults in the household has been reduced to an extent that implies that adults have repeatedly experienced the physical sensation of hunger (moderately food insecure).

**Severely food insecure (SFI)** - is all households with children who have reduced the children’s food intake to an extent indicating that the children have experienced hunger (food insecure with hunger) [46, 47].

### Sampling technique and sample size determination

Multistage cluster sampling (two stage cluster sampling) was employed. The East Gojjam zone was primarily classified into different clusters (districts) based on geographical or administrative characteristics, among which two districts were randomly selected. Second, the district was classified into Keble’s and some of the Keble’s were selected using simple random sampling technique (lottery method). Finally, households that are the smallest study unit were selected using systematic sampling technique.

Mathematical formulas for determining the sample size for ordinal outcome data were proposed [48], a non - parametric method based on the assumption of a constant odds ratio as given in Eq. 1.

1 $$ n=\frac{6\left[{\left({z}_{1-\raisebox{1ex}{$\alpha $}\!\left/ \!\raisebox{-1ex}{$2$}\right.}+{z}_{1-\beta}\right)}^2/{\left(\log \left(\theta\ \right)\right)}^2\right]}{\left(1-{\sum}_{i=1}^k{p_i}^3\right)} $$

Where n is the sample size required, *θ* is the odds ratio of households being in category j or less compared to the other, p_*i*_ is the proportion of households in category *i*, α is a level of significance, and 1 − β is power of the test. As computed from a pilot study, the proportions of households being in each category were found as *p*_1_ = 0.134, *p*_2_ = 0.198, *p*_3_ = 0.305 and  *p*_4_ = 0.363 with odds ratio of 2.852. Setting α = 0.01 and β = 0.1 the required sample size becomes 472.33. According to [37], since the number of categories is less than five, we multiply this sample size estimate by a correction factor c = 1.067 for i = 4, which becomes 504. Consequently, in this study, we considered 504 households.

Proportional Odds Model (POM)^1^ and Partial Proportional Odds Model^2^ were used as a tool to model the ordinal nature of our dependent variable (food insecurity status of households as highly food secure, marginally food secure, low food secure and severely food insecure, which has natural ordering pattern with the degree of severity increases) by defining the cumulative probabilities differently instead of considering the probability of an individual event [50].

## Results

### General characteristics of households

A total of 504 households from two districts, explicitly Machakel and Shebel Berenta, were considered in this study. The food insecurity status of households was determined from the 18-item Core Food Security Module (CFSM) question series designed by USDA, which is recognized as the standard measure of food insecurity in virtually all national, state and local surveys. The prevalence of food insecurity among households, 54 (10.71% CI: 8.29, 13.74) were highly food secure, 75(14.88% CI: 12.02, 18.27) were marginally food secure, 157 (31.15% CI: 27.24, 35.34) were low food secure and 218 (43.25% CI: 38.97, 47.63) were severely food insecure (Table 1).

**Table 1:** Prevalence of food insecurity status of households in the study area

Household Food Insecurity status	Count	Percent (95%: CI)
Highly Food Secure	54	10.71(8.29,13.74)
Marginally Food Secure	75	14.88 (12.02,18.27)
Low Food Secure	157	31.15(27.24, 35.34)
Severely Food Insecure	218	43.25(38.97, 47.63)

The prevalence of food insecurity varies by district which was 21.6, 19.1, 36.1 and 23.2% for Shebel Berenta, and 0.8, 11, 26.6 and 61.6% for Machakel had HFS, MFS, LFS, and SFI status. It shows that the proportion of highly food secure, marginally food secure, and low food secure status were higher in Shebel Berenta district, and to the contrary the proportion of severely food insecure was higher in Machakel district. Furthermore, the proportion of households across the status of gender and religion shows high prevalence as the severity level of food insecurity increase (Table 2). The prevalence of food insecurity indicates a difference in the household marital status, out of 405 married respondents, about 9.4, 15.6, 32.8 and 42.2% respectively had HFS, MFS, LFS and SFI status. The prevalence of being severely food insecure (SFI) slightly decreased as the level of education increase, with illiterate (50.6%), read and write (45.4%) and, more than high school (38.5%).

**Table 2:** Demographic characteristics of household head by food insecurity status of selected households, 2017

Characteristics	Food Insecurity level of Household				Total	Chi-square^P,F^ (*p*-value)
	Highly Food Secure	Marginally Food Secure	Low Food Secure	Severely Food Insecure		
	Count (%)	Count (%)	Count (%)	Count (%)		
District						
Shebel Berenta	52(21.6%)	46(19.1%)	87(36.1%)	56(23.2%)	241	102.7672 (0.000*)
Machakel	2(0.8%)	29(11.0%)	70(26.6%)	162(61.6%)	263	
Gender						
Male	42(10.4%)	62(15.3%)	127(31.4%)	174(43.0%)	405	0.5498 (0.908)
Female	12(12.1%)	13(13.1%)	30(30.3%)	44(44.4%)	99	
Religion						
Christian	54(11.1%)	71(14.6%)	152(31.2%)	210(43.1%)	487	2.8453 (0.416)
Muslim	0(0.0%)	4(23.5%)	5(29.4%)	8(47.1%)	17	
Marital status						
Married	38(9.4%)	63(15.6%)	133(32.8%)	171(42.2%)	405	27.7124 (0.001*)
Single	3(12.5%)	2(8.3%)	1(4.2%)	18(75.0%)	24	
Divorced	1(3.7%)	5(18.5%)	12(44.4%)	9(33.3%)	27	
Widowed	12(25.0%)	5(10.4%)	11(22.9%)	20(41.7%)	48	
Education status						
Illiterate	5(5.7%)	10(11.5%)	28(32.2%)	44(50.6%)	87	35.9127 (0.000*)
Read and write	29(8.8%)	41(12.5%)	109(33.2%)	149(45.4%)	328	
Elementary completed	14(22.2%)	18(28.6%)	16(25.4%)	15(23.8%)	63	
≥ High school	6(23.1%)	6(23.1%)	4(15.4%)	10(38.5%)	26	

*F* Fisher’s exact test*, P* Pearson’s chi-square

*indicates statistically significant at 5% level

Out of the 368 households who replied no saving practice habit, about 9.8, 13.9, 33.2, and 43.2% had HFS, MFS, LFS, and SFI status respectively. From 416 households that had not taken loan in the last 12 months, about 8.9, 13.9, 33.7, and 43.5% had HFS, MFS, LFS, and SFI status respectively (Table 3). On the other hand, 432 respondents of 504 had their own toilet access, which indicates the existence of good sanitation practices in the study area. In contrast, most households had low water treatment practices, and pipe and river were the main sources of drinking water (Table 3).

**Table 3:** Household food insecurity status in terms of socioeconomic and access for infrastructure characteristics

Characteristics	Highly Food Secure	Marginally Food Secure	Low Food Secure	Severely Food Insecure	Total	Chi-square^P,F^ (*p*-value)
	Count (%)	Count (%)	Count (%)	Count (%)		
Source of access to energy						
Kerosene	15(6.69%)	20(8.93%)	61(27.2%)	128(57.2%)	244	67.5912 (0.000)
Solar panels	12(9.1%)	28(21.2%)	49(37.1%)	43(32.6%)	132	
G. electric	21(18.3%)	23(20.0%)	43(37.4%)	28(24.3%)	115	
Other	6(18.2%)	4(12.1%)	4(12.1%)	19(57.6%)	33	
Habit of credit service (saving)						
Yes	18(13.2%)	24(17.6%)	35(25.7%)	59(43.4%)	136	3.8169 (0.282)
No	36(9.8%)	51(13.9%)	122(33.2%)	159(43.2%)	368	
Have you taken a loan, for the last 12 months						
Yes	17(19.3%)	17(19.3%)	17(19.3%)	37(42.0%)	88	13.6048 (0.003)
No	37(8.9%)	58(13.9%)	140(33.7%)	181(43.5%)	416	
Is toilet available						
Yes	47(10.9%)	63(14.6%)	126(29.2%)	196(45.4%)	432	7.2103 (0.065)
No	7(9.7%)	12(16.7%)	31(43.1%)	22(30.6%)	72	
Source of drinking water						
Pipe	43(13.4%)	53(16.5%)	98(30.5%)	127(39.6%)	321	1.2635 (0.62)
Pond	2(9.1%)	4(18.2%)	6(27.3%)	10(45.5%)	22	
River	5(3.6%)	16(11.6%)	48(34.8%)	69(50.0%)	138	
Other	4(17.4%)	2(8.7%)	5(21.7%)	12(52.2%)	28	
Do you treat water						
Yes	17(12.9%)	21(15.9%)	53(40.2%)	41(31.1%)	132	1.7073 (0.785)
No	37(9.9%)	54(14.5%)	104(28.0%)	177(47.6%)	372	

*F* Fisher’s exact test, *P* Pearson’s chi-square

*significant at 5% level of significance

In the present study, the fertility of agricultural land, irrigation practice, use of the improved seed, access to training on agricultural extension package, and use of fertilizer from each household were considered. Most households have had the opportunity to access fertilizer, and they have the opportunity to be trained by professionals on the agricultural extension package (Table 5). For instance, the prevalence of food insecurity shows a variation across household’s agro-ecological zone; nearly 20.6, 54.9, and 16.5% of households in kolla, Woina Dega, and dega agro-ecological zone had a severe food insecure status respectively. Majority of the respondents, about 417 households didn’t practice irrigation, which could contribute to improving the food security condition, and of that 12.9, 17, 29.7, and 40.3% had an HFS, MFS, LFS, and SFI status respectively.

Furthermore, the proportion of food insecurity status shows a variation in the slope of agricultural land. Most households around 384 of the respondents had a moderately sloped agricultural land, and among them 8, 11.6, 33.9, and 45.5% had an HFS, MFS, LFS, and SFI status respectively. On the other way, around 398 respondents had private land ownership with 11.6, 14.3, 30.9, and 43.2% had an HFS, MFS, LFS and SFI status respectively (Table 4).

**Table 4:** Agricultural environment and practice related characteristics of household by food insecurity level, 2017

Characteristics	Highly Food Secure	Marginally Food Secure	Low Food Secure	Severely Food Insecure	Total	Chi-square^P,F^ (*P*-value)
	Count (%)	Count (%)	Count (%)	Count (%)		
Agro ecological zone						
Kola	3(4.8%)	15(23.8%)	32(50.8%)	13(20.6%)	63	81.6389 (0.000)
Woina Dega	26(7.6%)	38(11.0%)	91(26.5%)	189(54.9%)	344	
Dega	25(25.8%)	22(22.7%)	34(35.1%)	16(16.5%)	97	
Slope of land						
Level	18(24.3%)	21(28.4%)	14(18.9%)	21(28.4%)	74	35.2571 (0.000)
Medium	31(8.0%)	49(12.7%)	131(33.9%)	176(45.5%)	384	
Gentle slope	5(11.6%)	5(11.6%)	12(27.9%)	21(48.8%)	43	
Land ownership						
Private	46(11.6%)	57(14.3%)	123(30.9%)	172(43.2%)	398	7.3372 (0.291)
Rented	6(7.0%)	13(15.1%)	25(29.1%)	42(48.8%)	86	
Collaborate	2(10.0%)	5(25.0%)	9(45.0%)	4(20.0%)	20	
Fertility of agricultural land						
Infertile	5(12.8%)	1(2.6%)	3(7.7%)	30(76.9%)	39	42.0617 (0.000)
Medium	41(9.4%)	65(14.9%)	149(34.1%)	182(41.6%)	437	
Fertile	8(28.6%)	9(32.1%)	5(17.9%)	6(21.4%)	28	
Irrigation practice						
Yes	0(0.0%)	4(4.6%)	33(37.9%)	50(57.5%)	87	25.2040 (0.000)
No	54(12.9%)	71(17.0%)	124(29.7%)	168(40.3%)	417	
Do you use improved seed						
Yes	41(9.1%)	65(14.4%)	142(31.6%)	202(44.9%)	450	13.4292 (0.004)
No	13(24.1%)	10(18.5%)	15(27.8%)	16(29.6%)	54	
Training by agricultural profession						
Yes	46(9.9%)	70(15.1%)	141(30.5%)	206(44.5%)	463	6.3471 (0.096)
No	8(19.5%)	5(12.2%)	16(39.0%)	12(29.3%)	41	
Fertilizer use						
Yes	49(10.6%)	71(15.4%)	143(31.0%)	199(43.1%)	462	1.0560 (0.788)
No	5(11.9%)	4(9.5%)	14(33.3%)	19(45.2%)	42	

*F* Fisher’s exact test, *P* Pearson’s chi-square

*significant at 5% level of significance

In the present study, the mean monthly income of household for HFS, MFS, LFS, and SFI households was ETB 965.01 ± 774.08, 724.59 ± 560.85, 804.54 ± 791.74, and 741.62 ± 409.99 respectively, and comparatively SFI households have low monthly income in typical. The mean landholding in hectare of households for HFS, MFS, LFS, and SFI status was 1.71 ± 0.88, 2.015 ± 1.07, 1.86 ± 1.1, and 1.66 ± 0.86 respectively. The mean family size of households for HFS, MFS, LFS, and SFI status was 4.8 ± 2.14, 4.2 ± 1.56, 4.5 ± 1.78, and 4.78 ± 1.92 respectively. The mean TLU of the household for HFS, MFS, LFS, and SFI status was 4.23 ± 1.62, 2.87 ± 1.47, 2.83 ± 2.17, and 3.2 ± 2.66 respectively, comparatively SFI households have low livestock in average (Table 5).

**Table 5:** Summary statistics showing the prevalence of different levels of food insecurity among households across continuous predictors and Univariate analysis

Predictor	HFS		MFS		LFS		SFI		*P*
	Mean ± SD		Mean ± SD		Mean ± SD		Mean ± SD		
Age	49.75	14.66	44.9	12.43	44.83	13.93	46.03	13.65	0.576
Family size	4.814	2.137	4.28	1.555	4.504	1.78	4.775	1.924	0.188*
Land size	1.706	0.886	2.015	1.078	1.861	1.102	1.668	0.862	0.055**
Monthly income	965.01	774.08	724.59	560.85	804.54	791.74	741.62	409.99	0.145*
Monthly Expenditure	1905.85	2260.51	974.52	1226.48	1110.83	6806.88	610.11	701.93	0.225
TLU	4.23	1.62	2.87	1.47	2.83	2.17	3.2	2.66	0.390

*SD* Standard deviation, *HFS* Highly food secure, *MFS* Moderately food secure, *LFS* Low food secure, *SFI* Severely food insecure;

**Significant at 10% and *Significant at 20% level of significance

### Specification of empirical model

The ordinal logistic regression model was selected to analyze food insecurity data by incorporating a set of predictors associated with the dependent variable, food insecurity status. To address all factors that are believed to affect the status of food insecurity appropriately, we reviewed factors that were demonstrated in previous related studies.

### Univariate analysis

For screening important associated food insecurity predictors, the association of each explanatory variables with the response using Pearson and Fisher exact chi-square test for the categorical predictors (Tables 3, 4 and 6) and univariate fit (Table 5) of POM for the continuous predictors were performed. As a result, POM was fitted together with explanatory variables that appear to be important in univariate analysis [51]. Furthermore, TLU was a statistically insignificant, but it is an economically important predictor that is directly related to the households ‘food insecurity status [37, 52–54] based on [51, 55] which supports these variables to be included.

**Table 6:** Overall all tests of parallel regression assumption (proportionality assumption tests for multivariable POM

Tests	Chi2	df	P > Chi2
Wolfe Gould	164.6	52	0.000
Brant	211.1	52	0.000
Score	155.9	52	0.000
Likelihood ratio	204.2	52	0.000
Wald	93.02	52	0.000

### Multivariable results of proportional odds model (POM)

The multivariable proportional odds model was fitted for the explanatory variables that appear to be important at univariate analysis with food insecurity level. Maarten Bui’s oparallel command was employed with the fitted proportional odds model to see if the fitting of a multivariable proportional odds model is appropriate for the data [56]. This command performs five tests a likelihood ratio test, a score test, a Wald test, a Wolfe-Gould test, and a Brant test (Table 6). All tests indicate that our data breached the proportional odds assumption; as a significant test statistic, there is evidence that the parallel regression assumption or proportional odds assumption has been violated. A violation of this assumption indicates that the effects of one or more independent variables significantly vary across cut point equations in the model [57].

In practice, when the proportional odds assumption is violated or difficult to achieve in practice by the data, the standard advice in such situations is to use the Partial proportional odds model (PPOM) which is more parsimonious and interpretable than those fitted by a non-ordinal method [58]. In PPOM, the assumption of parallel lines or proportional odds can be relaxed for some explanatory variables while being maintained for others.

### Multivariable partial proportional odds model (MPPOM)

A Multivariable partial proportional odds model with logit function was fitted (Table 7). For the final model, a global Wald test was performed with restricted (constrained) versus the original unconstrained model. The test indicates that the final model does not violate the assumption of parallel lines. As the global Wald test shows, in the final model, ten constraints were imposed, the chi2 (10) = 12.22 with *P*-value = 0.2705 being insignificant test statistics indicates that the final model does not violate the proportional odds or parallel line assumption.

**Table 7:** Multivariable PPOM analysis on associated factors of food insecurity adjusted odds ratio and CI estimates

Predictors	Categories	Model 1		Model 2		Model 3	
		Odds ratio (95% CI)	*P*-value	Odds ratio (95% CI)	*P*-value	Odds ratio (95% CI)	*P*-value
District	Machakel	104.2 (12.17,82,845.1)	0.000**	1.119 (0.481,2.60)	0.792	3.28(1.73,6.24)	0.000**
Marital status^a^	married	.002(6.70e-14,6.44e-07)	0.616	0.74(0.118,4.62)	0.748	14.4(3.36,61.58)	0.000*
	Divorced	.037(0.00007, 1.188)	0.065	1.81(0.514,6.34)	0.355	1.13(.36,3.49)	0.833
	widowed	.119(.0015, 1.094)	0.081	1.558(0.55,4.39)	0.401	2.53(0.026,6.21)	0.064
Family size		0.817(0.416,1.827)	0.0712	1.23(1.012,1.49)	0.037*	1.18(0.01,1.36)	0.083
Educatio status	Read and write	169.29(11.64, 2461.39)	0.000**	0.6712(.29,1.52)	0.342	1.31(.68,2.51)	0.422
	Elementary	119.75(8.43,1700.74)	0.000*	0.4422(.161,1.21)	0.113	0.69(0.26,1.83)	0.458
	Illiterate	113.4091(7.02,1832.02)	0.001*	0.383(.099,1.48)	0.164	1.77(0.50,6.26)	0.375
Ecological zone	Woina Dega	0.0021(.00009,.0514)	0.000*	1.82 (0.81,4.127)	0.147	4.75(0.98,11.36)	0.082**
	Dega	0.0323(.002,.5209)	0.016*	0.763(0.29,1.9827)	0.579	1.55(0.52,4.62)	0.431
Landholding		0.767(.605,0.972)	0.028*	0.766(0.61,.97)	0.028	0.76(0.60,0.97)	0.028*
Slope^a^	medium	0.58(.025,1.597)	0.091	2.74(0.24,6.09)	0.113	0.89(0.40,2.01)	0.794
	Gentle slope	1.53 (0.14,816.84)	0.075	2.88 (.89,9.34)	0.077	1.07(0.36,3.13)	0.903
Access to energy	G-electric	3.98 (0.914,70.73)	0.082	0.243(0.107,0.55)	0.001	0.468(0.23,.94)	0.033*
	solar panels	5.99 (0.039,125.50)	0.085	0.427(.202,0.906)	0.027	0.45(0.25,0.79)	0.006**
Soil fertility	medium	4.407 (0.92,21.106)	0.063	1.73(.46,6.45)	0.414	0.28(0.12,0.87)	0.010*
	fertile	11.23 (1.363, 92.59)	0.025*	1.073(.197,5.85)	0.934	0.15 (0.032,0.72)	0.017*
M.Income^a^		1.0002(0.99,1.0005)	0.550	1.0001(0.99,1.00)	0.550	1.00(0.99,1.00)	0.550
Loan status	No	0.484(0.1153, 2.028)	0.321	2.83(1.36,5.89)	0.006**	1.06(0.564,2.01)	0.844
Toilet access	No	7.63(1.459702,39.78)	0.016*	1.119(0.53,2.37)	0.768	0.77(0.39,1.54)	0.472
Seed I.^a^	No	0.84(0.403946, 1.720)	0.623	0.833(.404,1.72)	0.623	0.83(0.403,1.72)	0.623
Training^a^	No	0.92(0.397, 2.110)	0.836	0.92 (0.397,2.11)	0.836	0.92(.397,2.11)	0.836
Irrigation	Yes	0.00001(1.85e-06, 0.0001)	0.000**	0.121(.037,0.38)	0.00**	0.94(.505,1.75)	0.850
TLU		0.151(0.0716,0.3189)	0.000**	0.920 (0.79,1.07)	0.286	0.99(0.884,1.10)	0.804

‘*’ and ‘**’ statistically significant at 5 and 1% level of significance, Model 1: HFS Vs. MFS, LFS, SFI; Model 2: HFS, MFS, Vs. LFS, SFI; Model 3: HFS, MFS, LFS, Vs. SFI, ^a^variables that were not significantly associated with food insecurity have been presented in the table

### Test of overall model fit and goodness of fit test

The better - fit model was found among the ordinal logistic regression models considered in this study as a multivariable partial proportional ordinal model, with AIC of 993.5806. Besides, the full PPOM model was compared to the null model (only intercept model) using the likelihood ratio test (LRT) which tests whether the current model predicts better than the intercept only model. The value of the likelihood ratio chi-square statistic is *LR* (68) = 407.04 with P-value = 0.0000 implies that the full model predicts the data better than the intercept only model (Table 8). The Hosmer and Lemeshow test was conducted on a series of binary logistic regression model to check the goodness of fit. All the Hosmer and Lemeshow test of the series of the binary logistic regression model are insignificant, showing that the three binary logits fit the data well (Table 9).

**Table 8:** Likelihood ratio test for PPOM

Model	obs	Log likelihood		df	Information criterion’s		Likelihood ratio test	
		ll (null)	ll (model)		AIC	BIC	LR chi2	Prob > chi2
PPOM	504	− 629.3112	− 425.7903	71	993.5806	293.38	407.04	0.0000

**Table 9:** Goodness of fit test for SBLM

Separated binary logit model (SBLM)	Chi-square	df	Sig.
HFS Vs. MFS and LFS and SFI	12.752	8	.121
HFS and MFS Vs. LFS and SFI	14.379	8	.072
HFS and MFS and LFS Vs. SFI	10.028	8	.263

In the present study, various predictors expected to be the determinants of the households’ food insecurity status were considered. The multivariable PPOM analysis on socio-economic, demographic, institutional, agricultural and institutional characteristics showed that district, HH marital status, family size, HH education status, ecological zone, agricultural land size (hec.), source of access to energy, soil fertility, loan status, toilet access, irrigation practice, and Tropical Livestock Unit (TLU) were found to be associated factors for food insecurity of households in the study area (Table 7).

## Discussions

This study assesses the prevalence and food insecurity associated factors in rural households in selected districts of East Gojjam Zone, North West Ethiopia. Accordingly, out of 504 respondents, the prevalence of food insecurity was found to be 10.71, 14.88, 31.15, and 43.25% as highly food secure, marginally food secure (food - insecure without hunger), low food secure (moderately food insecure), and severely food insecure (food insecure with hunger) respectively (Table 1). The observed food insecurity prevalence report is high, which may be due to the fact that most households were unable to produce adequate food at the household level, low power of commonly produced agricultural commodities in generating income, lack of government subsidies and inappropriate distribution, and an intervention related to a food security program should be addressed in the study areas. This result shows a slim difference or inconsistency with the study [53] conducted in Borana Zone, Oromia, Ethiopia. His result of prevalence showed that 23, 25, 31, and 21% of respondents were food secure, food insecure without hunger, food insecure with moderate hunger, and food insecure with severe hunger, respectively. This difference shows that there is a discrepancy in the prevalence of food insecurity in different neighborhoods within the country.

The risk of food insecurity shows a variation across districts in this finding. Comparing households under HFS and MFS and LFS to SFI, households belonging to machakel district were more likely to be severely food insecure (AOR = 3.28, 95% CI: 1.73, 6.24, *P* < 0.001) as compared to households belonging to Shebel berenta district (Table 7). This might be because of low agricultural production in the machakel district due to deprived soil fertility, low livestock production, land shortages, continuing cash income shortages, poor farming technologies, and weak extension services than the Shebel berenta district. This results in proof of concern that food insecurity is a multidimensional phenomenon faced differently by different types of households and population groups [45, 59].

The household head education achievement was found to be a statistically significant associated factor for households ‘food insecurity status. In this study, various forms of household head education status were tested to investigate their effect on household food insecurity status, including illiterate, read and write, elementary completed, and high school and above. The result showed that when HFS status was compared with MFS, LFS and SFI status, households having household head education status with illiterate, read and write, and elementary completed level of education were more likely (AOR =113.4, 95% CI:7.02,1832.02,*P* = 0.001; AOR = 169.29, 95%CI:11.64,2461.39, *P* < 0.001, AOR = 119.75, 95%CI: 8.43, 1700.74, *P* < 0.001 respectively) to be MFS, and LFS, and SFI status than households who have high school and above education status. This finding shows that the head of households with a low level of education had a higher risk of being food insecure (Table 7). This result is supported by studies done in Punjab Pakistan, Ethiopia’s Central Zone of Tigrai [37, 41]. This could be explained by education as a tool for accessing information on best agricultural production, nutrition, and sanitation increased efficiency through enhanced manufacturing and better decision-making. Then education played an active part in improving the capacities of people that can generate a beneficial element in the fight against food insecurity.

The ecological zone was found to be a significant factor associated with household food insecurity. Households living in Woina Dega and Dega were less likely to be in MFS and LFS or SFI status (as opposed to HFS status) compared to households living in Kolla agro-ecological zone (AOR = 0.0021,95% CI: 0.00009,.0514,*P* < 0.000; 0.0323, 95%CI: 0.002,.5209, *P* = 0.016) respectively (Table 7). This result is supported by the study conducted in Ethiopia by [60]. This may be due to the fact that high production failures in the kola agro-ecological zone due to frequent droughts (less rainfall), so that they are unable to diversify food access through high dependence of food purchases rather than production. This study suggests that agricultural extension should take water conservation into account and extend irrigation practices to cope with less agricultural - productive areas, especially for the kola agro-ecological zone.

The finding of this study indicates having a large family size creates more pressure on household food security. For one unit increase in family size, the chances of being food insecure were 18% higher (AOR = 1.18, 95%CI: 1.01, 1.36, *P* = 0.034) than the lower status of food insecurity HFS, MFS, and LFS (Table 7). This finding is supported by the studies done in Nigeria, Ethiopia, Pakistan, and South Africa [61–65]. This may be due to the fact that larger families have a higher chance of possessing a lower income per capita and a high need for more food availability and more resources to buy food [66, 67]. These finding encourages for implementing community-based Education that may lead to lower birth rates, and therefore reducing family size*.*

In this study, landholding size in hectare of the household was found to be a significant associated factor for the household’s food insecurity status. The odd ratio of the highest category of food insecurity (severely food insecurity) versus the highly food - secure and marginally food - secure and low food - security status was 23.3% lower as opposed to the other category (AOR = 0.767, 95% CI: 0.605, 0.972, *P*-value =0.028), given that the other variables in the model kept constant (the odds of food insecurity would be reduced by 23.3% for one unit increase in the landholding of households) (Table 7). This may be due to the fact that access for a large landholding leads to high opportunities for agricultural production, aggregate tree cultivation practices that can serve as a system of income generation, and sufficient access for livestock grazing. This finding is consistent with the study results reported from Ethiopia by [65]. This study encourages that due to extremely limited land ownership in Ethiopia, a mixed farming system (integrated elements of both livestock and crop cultivation) should be adopted on the small available land to reduce the prevalence of food insecurity.

Comparing HFS and MFS and LFS with SFI status, households who had access to energy in the form of government electric and solar panels were less likely (AOR = 0.468, 95% CI: 0.23, 0.94, *P* = 0.033; AOR = 0.45, 95% CI: 0.25, 0.79, *P* = 0.006) to be severely food insecure compared to households with access to energy are kerosene lamps (Table 7). This may be due to the fact that poor access to energy such as kerosene lamps and fuel wood leads to reallocation of household resources from food production and preparation to fuel procurement, potentially affecting cooking practices and dietary choices. This finding suggests that access to energy in the form of renewable energy has its own contribution to reducing the prevalence of food insecurity for rural households. So the government and concerned bodies should pay attention to renewable energy such as electric and solar panels to improve food security for rural households.

Agricultural soil fertility was found to be a significant associated factor for food insecurity. When comparing HFS and MFS and LFS households with SFI status, households with medium and fertile soil fertility status were less likely to be severely food insecure (AOR = 0.28, 95% CI: 0.12, 0.87, *P* = 0.010; AOR = 0.15, 95% CI: 0.032, 0.72, *P* = 0.017 respectively) compared to households with infertile (poor fertility) soil (Table 7). This may be due to the fact that farming on soil with good soil fertility can increase agricultural productivity [68]. This study encourages giving attention to healthy soil on soil management, awareness of soil condition programs and soil productivity improvement program. In line with this farmer field schools such as cover crops that add organic matter to the soil, and green manure or growing legumes to fix nitrogen from the air over the biological nitrogen fixation process are highly recommended for increasing soil fertility.

The tropical livestock unit (TLU) which is the aggregate number of livestock converted to a common unit (livestock owned by the head of the household) was found to be a significant associated factor for household food insecurity. When HFS was compared with MFS and LFS and SFI status, for one unit increase in TLU, the odds of being food insecure would decrease by 84.8% (AOR = 0.15195% CI: 0.0716, 0.3189, *P* < 0.001) (Table 7). This may be due to the fact that, currently in Ethiopia there is an increasing demand for animal products in the growing cities. In addition, the increasing populations hardly need livestock and their products, such as meat and milk, for consumption and trade, which serve as a means of generating income by selling these livestock when confronted with food shortages. This result is consistent with the studies [37, 40, 53] conducted in Ethiopia. As a result, the concerned body must devise approaches with a high level of attention to increase the production of livestock in the study area, as livestock is a driving force for food security.

The other predictor found to be a significant associated factor for food insecurity in this finding was access to the toilet. When comparing HFS with MFS and LFS and SFI status, households with no access to the toilet were more likely (AOR = 7.63, 95% CI: 1.459, 39.78, *P* = 0.016) to be MFS and LFS and SFI as compared to households with access to the toilet (Table 7). This could be due to the fact that food security explained by access to sanitation, then access to the toilet for rural households improves food security. This finding is supported by [69] factors such as knowledge and practices in nutrition and access to quality health care, clean water and sanitation are crucial for ensuring food security. The result recommended to the healthcare workers to focus on hygiene education or shift in sanitation practice (for sustainable sanitation) with a renewed alliance between the healthcare worker and community leaders.

According to this study, irrigation practice was another associated factor of food insecurity in the household. When HFS and MFS were compared with LFS and SFI status, households practicing irrigation were less likely (AOR = 0.121, 95% CI: 0.037, 0.38, *P* = 0.00) to be low food secure and severely food insecure as compared to households not practicing irrigation(Table 7). This could be because improved water management and use are fundamental to increasing agricultural yields, and the link between water services for irrigation opportunities for individuals and communities lifts food production, both in quantity and diversity, to satisfy the basic needs of households and also to generate surplus income. As supported by other studies [70, 71], the efficient use of available irrigation water is an important concern to improve food security. This study enforces the government to pay due attention to small - scale irrigation systems managed by the community that is effective in alleviating rural poverty and eradicating food insecurity by improving yields and crop intensities.

When HFS and MFS compared with LFS and SFI status, households that do not take a loan from any financial institution were more likely (AOR = 2.83, 95% CI: 1.36, 5.89, *P* = 0.006) to be low food secure and severely food insecure compared to households that take a loan from financial institutions (Table 7). This indicates that the risk of being food insecure for households who do not take a loan was higher than households who take a loan from financial institutions. This could be due to the fact that households receiving a loan from a financial institution can increase household income by purchasing livestock, inputs for agricultural activities, and make farming households allocate labor more efficiently, which increases the well - being of farming households as well as agricultural outputs. This finding supports the idea of access to finance as a critical piece of food security [11] by increasing household income leading to an increase in household food availability.

## Conclusions

The findings from this study show a high prevalence of food insecurity in the study area and different associated food insecurity factors have been identified. Thus, interventions by the bodies concerned on food insecure households should ruminate water conservation and extend irrigation practices to cope with less agricultural-productive areas, especially kola agro-ecology zone, and community-based education to access information on best agricultural production, nutrition, and sanitation, as well as lower birth rates, which can reduce family size. As TLU and landholding are significant associated factors of food insecurity, attention should be given to increase livestock production and mixed farming on the small available landholding to reduce the prevalence of food insecurity. Besides, healthy soil would be accomplished through soil management and awareness of soil condition programs and soil productivity improvement programs through farmer field schools. Finally, further study should be conducted to identify mechanisms for addressing food insecurity in the study area that will be adopted for rural households.

## Supplementary information

**Additional file 1.** USDA core food security modules (CFSM) question series as a Guideline for measuring the food insecurity status of households in selected districts of East Gojjam Zone, Ethiopia, 2017.

## Data Availability

The primary data set collected from households and analyzed during the current study is available from the corresponding author.

## Abbreviations

AIC: Akaike’s information criterion
BIC: Bayesian information criterion
CFSM: Core food security module
FAO: Food and Agriculture Organization
HFS: Highly food secure
HH: Head of household
LFS: Low food secure
MDG: Millennium Development Goal
MFS: Marginally food secure
POM: Proportional odds model
PPOM: Partial proportional odds model
SBLM: Separated binary logistic regression model
SFI: Severely food insecure
TLU: Tropical livestock unit
USDA: United state Department of Agriculture
WFS: World Food Summit

## Footnotes

1 Proportional odds Model(POM) Consider a collection of K independent variables (predictors) denoted by the vector *X*^′^ = [*X*_1_, *X*_2_, …, *X*_*K*_]. The relationship between the predictors and response variable is not a linear function in logistic regression, instead, the logistic regression function uses, the logit transformation of *π*_*i*_ given by:

2 $$ {\uppi}_{\mathrm{i}}=\frac{\exp \left[{\upalpha}_{\mathrm{i}}+{\upbeta}_1{\mathrm{X}}_1+{\upbeta}_2{\mathrm{X}}_2+\dots +{\upbeta}_{\mathrm{K}}{\mathrm{X}}_{\mathrm{K}}\right]}{1+\exp \left[{\upalpha}_{\mathrm{i}}+{\upbeta}_1{\mathrm{X}}_1+{\upbeta}_2{\mathrm{X}}_2+\dots +{\upbeta}_{\mathrm{K}}{\mathrm{X}}_{\mathrm{K}}\right]}\kern18.25em $$

Then the logit or log-odds of having pr(Y ≤ i) = π_i_ is modeled as a linear function of the explanatory variables as:

3 $$ \log \left[\frac{\mathrm{pr}\left(\mathrm{Y}\le \mathrm{i}\right)}{1-\mathrm{pr}\left(\mathrm{Y}<\mathrm{i}\right)}\right]=\log \left[\frac{\uppi_{\mathrm{i}}}{1-{\uppi}_{\mathrm{i}}}\right]={\upalpha}_{\mathrm{i}}+{\upbeta}_1{\mathrm{X}}_1+{\upbeta}_2{\mathrm{X}}_2+\dots +{\upbeta}_{\mathrm{K}}{\mathrm{X}}_{\mathrm{K}} $$

Which is equivalent with:

4 $$ \mathrm{logit}\left[\mathrm{pr}\left(\mathrm{Y}\le \mathrm{i}\right)\right]={\upalpha}_{\mathrm{i}}+\sum \limits_{\mathrm{j}=1}^{\mathrm{k}}{\upbeta}_{\mathrm{j}}{\mathrm{X}}_{\mathrm{i}},\kern0.5em \mathrm{i}:1,2,\dots, \mathrm{c}-1\kern0.5em \&\kern0.5em \mathrm{j}:1,2,\dots, \mathrm{k} $$

Equation (4) is called proportional odds model and it estimates simultaneously multiple equations of cumulative probability, each logit has its own ***α***_***i***_ term that is the threshold value and their values do not depend on the values of the independent variable for a particular case, and the coefficient ***β***_***j***_ indicates the direction and strength of the relationship between independent variable and the log odds of the dependent variable. For fitting an ordinal logistic regression using the proportional odds model the assumption is that the relationship between the independent variables across each logit is the same. When the proportional odds assumption is violated by the data, the partial proportional odds models (PPOM) will be employed, by relaxing parallel lines or proportional odds assumption for some predictors while being maintained for others.

2 Partial proportional Odds Model The partial proportional odds model (PPOM) formulated by Peterson and Harrell [49], which imposes constraints for parallel lines only where they are needed. The equation can be expressed as:

5 $$ \mathit{\ln}\left(\frac{\mathit{\Pr}\Big(y\le j\mid \boldsymbol{x}}{\mathit{\Pr}\Big(y>j\mid \boldsymbol{x}}\right)={\alpha}_j+\left(\boldsymbol{x}\beta +\boldsymbol{T}{\gamma}_j\right),\kern1.25em j=1,\dots, J-1 $$

Here **x** is the vector containing the full set of independent variables that satisfies the proportional odds assumption, ***T*** is a vector containing a subset of independent variables that violate the parallel assumption, *β* and *γ*_*j*_ are the regression coefficients associated with the variables in **x** and ***T*** respectively*.* The model given in equation (5) can be expressed in terms of logit as:

6 $$ \mathrm{logit}\left[\Pr \left(\mathrm{y}\le \mathrm{j}|\mathrm{x}\right)\right]={\upalpha}_{\mathrm{j}}+\sum \limits_{\mathrm{k}=1}^{\mathrm{m}}{\upbeta}_{\mathrm{k}}{\mathrm{x}}_{1\mathrm{k}}+\sum \limits_{\mathrm{r}=1}^{\mathrm{p}}{\upgamma}_{\mathrm{r}\mathrm{j}}{\mathrm{T}}_{2\mathrm{r}}\kern0.75em \mathrm{j}=1,\dots, \mathrm{J}-1\Big) $$
