# Supplementary material for: Prevalence and associated factors for rural households food insecurity in selected districts of east Gojjam zone, northern Ethiopia: cross-sectional study
Source: BMC Public Health. 2020 Feb 7;20:202. doi: 10.1186/s12889-020-8220-0 (PMC7007667; doi:10.1186/s12889-020-8220-0)
Supplement: Supplementary file 1 — Additional file 1. USDA core food security modules (CFSM) question series as a Guideline for measuring the food insecurity status of households in selected districts of East Gojjam Zone, Ethiopia, 2017. [file 12889_2020_8220_MOESM1_ESM.docx]

**Additional file 1:** USDA core food security modules (CFSM) question series as a Guideline for measuring the food insecurity status of households in selected districts of East Gojjam Zone, Ethiopia, 2017

|  | **Questions** | Response |
| --- | --- | --- |
| 1 | (I/We) worried whether (my/our) food would run out before (I/we) got money to buy more. | Often true  Sometimes true  Never true |
| 2 | The food that (I/we) bought just didn't last, and (I/we) didn't have money to get more. | Often true  Sometimes true  Never true |
| 3 | (I/we) couldn't afford to eat balanced meals. | Often true  sometimes true  Never true |
| 4 | (I/we) relied on only a few kinds of low-cost food to feed (my/our child/the children) because (I was/we were) running out of money to buy food. | Often true  Sometimes true  Never true |
| 5 | Did (you/you or other adults in your household) ever cut the size of your meals or skip meals because there wasn't enough money for food? | Yes  No |
| 6 | (I/we) couldn't feed (my/our child/the children) a balanced meal, because (I/we) couldn't afford that. | Often true  Sometimes true  Never true |
| 7 | Did you ever eat less than you felt you should because there wasn't enough money for food? | No  Yes |
| 8 | How often did (you/you or other adults in your household) cut the size of your meals or skip meals because there wasn't enough money for food? | Only 1–2 months  Some but not every  Almost every month |
| 9 | (My/Our child was/the children were) not eating enough because (I/we) just couldn't afford enough food. | Often true  Sometimes true  Never true |
| 10 | Were you ever hungry but didn't eat because you couldn't afford enough food? | Yes  No |
| 11 | Did you lose weight because you didn't have enough money for food? | Yes  No |
| 12 | Did you ever cut the size of (your child's/any of the children's) meals because there wasn't enough money for food? | Yes  No |
| 13 | Did (you/you or other adults in your household) ever not eat for a whole day because there wasn't enough money for food? | Yes  No |
| 14 | (Was your child/Were the children) ever hungry but you just couldn’t afford  more food | Yes  No |
| 15 | How often did (you/you or other adults in your household) not eat for a whole day because there wasn't enough money for food? | Only 1–2 months  Some but not every  Almost every month |
| 16 | Did (your child/any of the children) ever skip a meal because there wasn't enough money for food? | Yes  no |
| 17 | How often did (your child/any of the children) skip a meal because there wasn't enough money for food? | Only 1–2 months  Some but not every  Almost every month |
| 18 | Did (your child/any of the children) ever not eat for a whole day because there wasn't enough money for food? | Yes  No |

Source: <https://www.ncbi.nlm.nih.gov/books/NBK206920/>
